# Quantification of left and right atrial kinetic energy using four-dimensional intracardiac magnetic resonance imaging flow measurements

**DOI:** 10.1186/1532-429X-15-S1-P218

**Published:** 2013-01-30

**Authors:** Per Arvidsson, Johannes Toger, Einar Heiberg, Marcus Carlsson, Hakan Arheden

**Affiliations:** 1 Department of Clinical Physiology, Institution for Clinical Sciences, Lund, Sweden

## Background

Kinetic energy (KE) of atrial blood has been postulated as a possible contributor to ventricular filling. Furthermore, KE is independent of blood pressure and may thus be altered in disease with normal blood pressure. Atrial blood KE, however, has not previously been measured over the whole cardiac cycle, and thus its contribution to cardiac function remains unknown. We therefore aimed to quantify the left and right atrial blood KE using cardiac magnetic resonance (CMR), and to identify mechanisms contributing to atrial KE.

## Methods

Nine healthy volunteers underwent CMR, including a four-dimensional phase contrast flow sequence using a 3T MRI scanner. Atrial anatomy was manually delineated and KE within these delineations was calculated as KE = (m*v^2^)/2.

## Results

Mean left atrial (LA) KE was 1.1±0.1 mJ (mean±SEM), and mean right atrial (RA) KE was 1.7±0.2 mJ (P<0.01). Three KE peaks were seen in both atria; one in systole, one during early diastole, and one during atrial contraction. The systolic LA peak was significantly smaller than the RA peak (P<0.01). Early and late diastolic peaks did not differ, however the increase of early diastolic KE from end-systolic KE was much higher in the LA (P<0.01). There was a high correlation between mean systolic KE and the combination of atrial volume and systolic velocity of the atrioventricular plane displacement (R^2^=0.84 for LA and R^2^=0.93 for RA). The diastolic KE of the LA correlated with LV mass (R^2^=0.44), however no such correlation was found in the right heart. Atrial KE did not correlate with body surface area.

## Conclusions

Our findings suggest that LA KE increases during early diastole due to LV elastic recoil, indicating that LV filling is dependent on diastolic suction. RV relaxation does not seem to contribute to atrial KE. Instead, atrial KE generated during ventricular systole may contribute to RV filling during early diastole.

## Funding

This study was supported by grants from the Swedish Research Council, Sweden (2008-2461, 2008-2949, 2011-3916), National Visualization Program and Knowledge Foundation, Sweden (2009-0080), the Swedish Heart and Lung Foundation, Sweden, the Medical Faculty at Lund University, Sweden, and the Region of Scania, Sweden.

**Figure 1:**
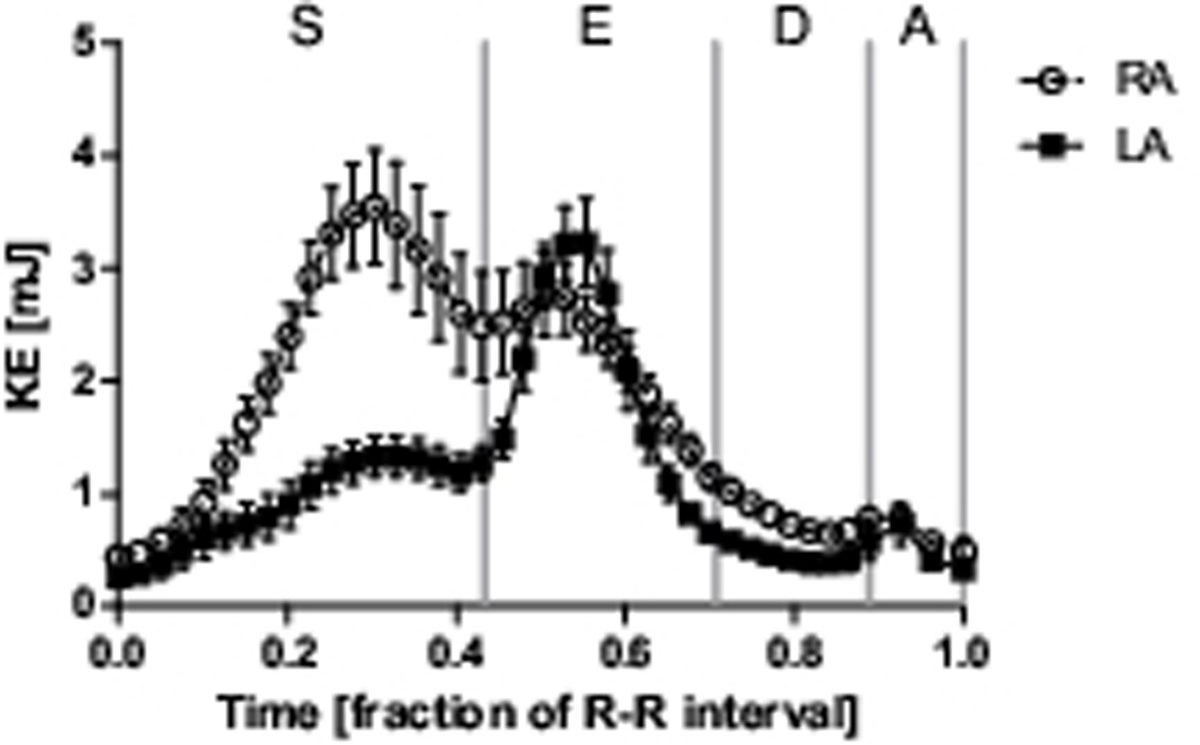
Mean left atrial (LA) and right atrial (RA) kinetic energy (KE) over the cardiac cycle for all subjects. Error bars indicate SEM. Gray vertical lines differentiate between systole (S), early diastole (E), diastasis (D), and atrial contraction (A), respectively. Three energy peaks are seen: 1) systole, 2) early diastole, and 3) late diastole. During systole, RA KE is significantly larger than LA KE. The early diastolic peaks were similar between both atria, however the increase in KE from end systole to early diastole was markedly larger in the LA. The late diastolic peaks were caused by atrial contraction, and did not differ between the atria.

**Figure 2:**
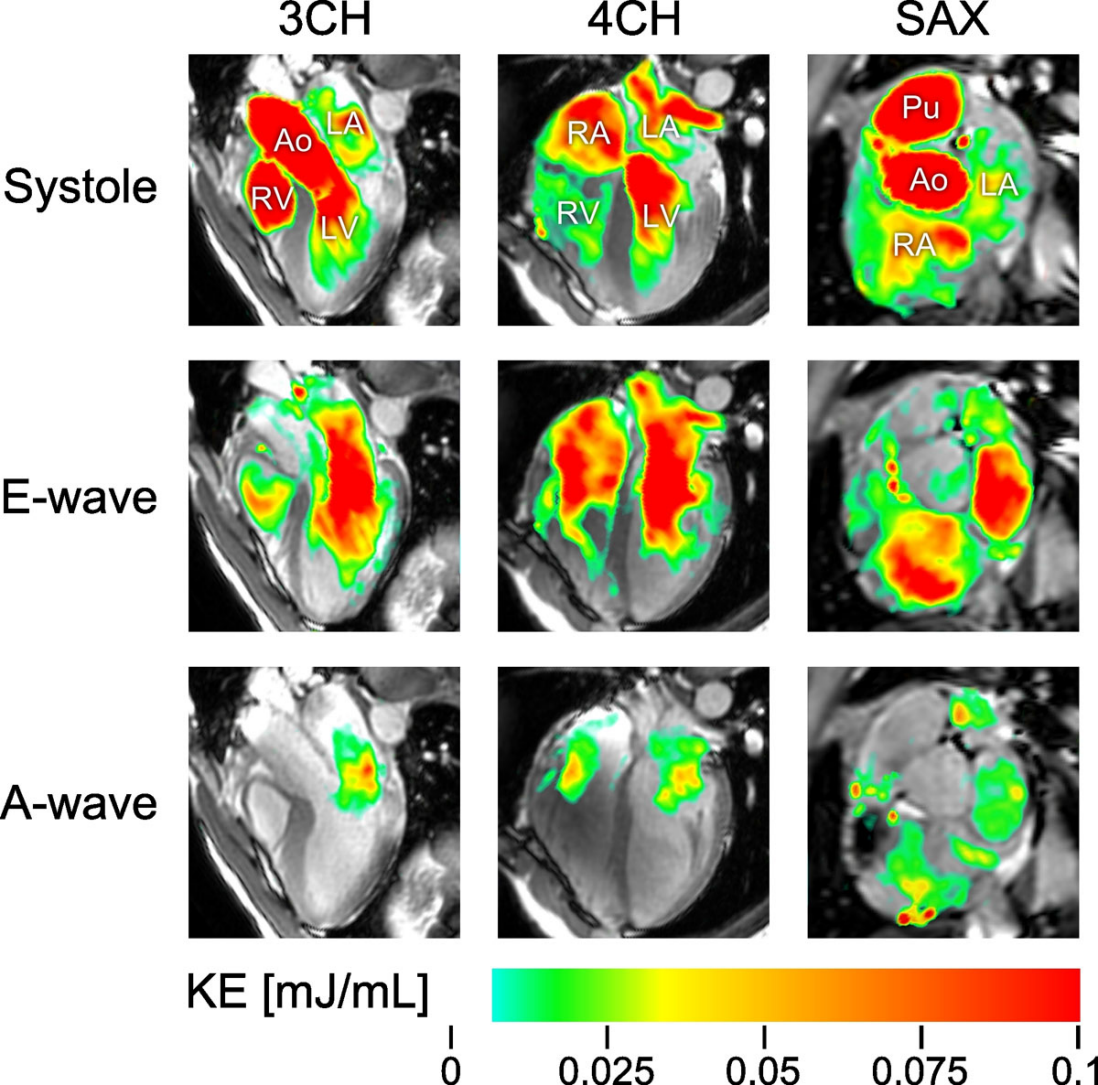
Visualization of the location and magnitude of kinetic energy (KE) in the heart of one representative volunteer at three time points during the cardiac cycle: at peak systole (top), at peak early diastolic filling (middle) and during atrial contraction (bottom). Three imaging planes are shown: three-chamber view (3CH, left), four-chamber view (4CH, middle), and a mid-atrial short-axis view (SAX, right). During systole, atrial KE is more prominent in the RA than in the LA, where KE is mainly located near the pulmonary vein inlets. Left atrial KE increases sharply during early diastolic filling, which is seen as a streak from the middle of the LA into the center of the LV. Meanwhile, RA KE also increases, but not as much. This increase in KE is likely caused by ventricular recoil. The KE during atrial contraction has a similar location as during early diastolic filling, but the magnitude is smaller.

